# Nursing leadership for sustainable endoscopy: a narrative review and practice framework

**DOI:** 10.3389/fmed.2026.1954846

**Published:** 2026-09-28

**Authors:** Peiying Zhang, Jinjin Qu, Chengmei Ge

**Affiliations:** Nursing Department, Sir Run Run Shaw Hospital, Zhejiang University School of Medicine, Hangzhou, China

**Keywords:** endoscopy nursing, nursing leadership, resource stewardship, sustainable endoscopy, waste segregation

## Abstract

Gastrointestinal endoscopy is a resource-intensive clinical service that generates substantial waste, consumes water and energy, and depends on disposable accessories, sterile packaging, and complex reprocessing pathways. Although green endoscopy has received increasing attention, current sustainability strategies have often emphasized physician decision-making, technological innovation, or industry-level redesign, while the practical contribution of endoscopy nurses remains insufficiently defined. This narrative review developed a nursing-centred framework for sustainable endoscopy practice, focusing on how nurses can translate green endoscopy policy into routine clinical action. Literature searches of PubMed/MEDLINE, Web of Science, Scopus, and Google Scholar were updated through 31 August 2026; CINAHL was subsequently searched on 4 September 2026, and professional-society statements and reference lists were hand-searched. The CINAHL search retrieved 403 records, with no unique additional records remaining after deduplication against the existing search set. Environmental evidence was classified as directly measured physical or resource outcomes, calculated or modelled carbon-footprint outcomes (including life-cycle assessment), or inferred downstream implications from clinical or operational findings. The evidence varied in study design, clinical setting, sustainability focus, and reported outcomes, and was synthesized thematically across the endoscopy pathway. Key domains included nursing-led waste segregation, consumable stewardship, cleaning and reprocessing support, patient preparation, and sustainability education, together with nursing-supported multidisciplinary decisions on device selection, procedure scheduling, and green procurement. Available evidence suggests that nursing-led interventions, including structured waste segregation, staff education, audit, careful management of consumables, and reprocessing stewardship, can reduce avoidable waste and, where carbon accounting was performed, associated calculated or modelled emissions and waste-processing costs while maintaining patient safety and procedural efficiency. However, nursing-specific intervention studies and standardized sustainability indicators remain limited, and much of the evidence originates from high-resource settings. A designated Green Endoscopy Nurse or sustainability-lead function may provide a useful implementation model for embedding environmental responsibility into everyday endoscopy nursing practice, although current evidence does not yet establish this role as a universal staffing standard.

## Introduction

Climate change has become a defining public health challenge for contemporary healthcare systems. Although healthcare services exist to protect and restore health, they also contribute to environmental degradation through greenhouse gas emissions, energy consumption, water use, chemical exposure, and waste generation. This creates an ethical and professional tension for nursing: clinical care must protect individual patients while also reducing avoidable harm to population and planetary health (1). Recent nursing scholarship has therefore argued that planetary health should be incorporated into nursing practice, education, leadership, and advocacy, rather than being treated as a peripheral environmental issue (2).

**Table 1:** Nursing-led and nursing-supported actions for sustainable endoscopy practice: professional role, evidence directness, and expected outcomes.

Nursing domain	Role classification	Specific nursing actions	Environmental evidence	Expected or reported outcome	Supporting evidence
Waste segregation and recycling	Nurse-led	Distinguish regulated medical waste from general waste; separate clean paper, cardboard, and plastic packaging before patient contact; place clearly labelled bins in procedure and preparation areas; conduct regular waste-stream audits.	Direct (waste mass, cost); calculated/modelled (kg CO2e)	Reduced regulated medical waste and processing costs; lower calculated emissions associated with waste treatment; improved segregation.	(16, 19, 20)
Consumable stewardship during procedure preparation	Nurse-led	Avoid premature opening of accessories; confirm procedure type and anticipated device needs before opening sterile packs; standardize but periodically review procedure trays; record unused opened items as part of quality improvement.	Direct (product/waste counts); carbon effect usually inferred unless separately modelled	Lower avoidable product use and waste; potential procurement savings; carbon effect requires separate modelling.	(12, 16, 41)
Cleaning and reprocessing stewardship	Nurse-led (within validated protocols)	Ensure timely bedside pre-cleaning; perform leak testing consistently; follow validated reprocessing protocols; monitor water and detergent use; avoid duplication of cleaning steps only where validated protocols permit.	Direct (water use, cleaning time); projected/modelled annual savings; carbon effect not always quantified	Lower water and chemical use, prolonged reusable endoscope lifespan, improved workflow efficiency.	(33, 35, 36)
Patient preparation and prevention of repeat procedures	Nurse-led	Provide nurse-led bowel preparation education; use telephone calls, written instructions, SMS reminders, or digital education tools; identify patients at high risk of inadequate preparation; reinforce preparation instructions during pre-procedure assessment.	Indirect (clinical outcome; environmental effect not directly measured)	Higher bowel preparation adequacy, fewer repeat colonoscopies, improved patient experience, and better procedural efficiency.	(10, 42)
Device selection: single-use versus reusable	Nurse-supported multidisciplinary (decision owner: endoscopist, infection prevention, procurement)	Participate in patient infection-risk assessment; reserve single-use devices for clearly justified cases under multidisciplinary policy; support safe reuse through correct pre-cleaning, documentation, and reprocessing compliance; report device damage or reprocessing barriers promptly.	Modelled (device-level LCA)	Context-dependent: reusable devices showed a lower per-procedure footprint under specific reprocessing conditions, while infection prevention justifies single-use devices in selected cases.	(10, 17, 18, 29)
Patient-flow and service redesign	Nurse-supported multidisciplinary (decision owner: endoscopist and unit management)	Coordinate same-day combined procedures when clinically appropriate; support virtual pre-assessment and post-procedure follow-up; reduce avoidable patient visits; ensure timely communication of preparation, medication, and follow-up instructions.	Modelled whole-procedure carbon footprint for same-day procedures; indirect for virtual care	Same-day EGD plus colonoscopy associated with a 42% lower combined calculated footprint in three US centers; virtual-care carbon benefit not directly quantified.	(7, 10, 45)
Green procurement and supplier engagement	Nurse-supported multidisciplinary (decision owner: procurement and unit leadership)	Involve nursing managers and Green Endoscopy Nurses in procurement discussions; prefer recyclable or lower-packaging consumables when clinically equivalent; request environmental information from suppliers; monitor high-use items and packaging burden.	Inferred	Lower packaging waste, improved product standardization, reduced overstocking, and stronger alignment between procurement and clinical workflow.	(7, 14)
Governance, education, audit, and the Green Endoscopy Nurse function	Nurse-led implementation within unit governance	Establish a Green Endoscopy Nurse or sustainability-lead function where feasible; integrate sustainability into orientation and annual training; monitor standardized indicators; report progress to unit leadership.	Direct process outcomes in selected interventions; calculated/modelled CO2e; implementation-role evidence limited	May facilitate sustained monitoring, feedback, and implementation; standardized indicators are increasingly available.	(15, 19, 20, 32)

'Direct’ refers to measured physical/resource/activity outcomes (e.g., waste mass, water, time, energy, or cost). Carbon footprints expressed as kg CO2e are calculated or modelled using emission factors or LCA, even when based on prospectively collected activity data.

The environmental impact of healthcare is substantial and measurable. A global assessment reported that healthcare contributes meaningfully to multiple environmental stressors, including greenhouse gas emissions, air pollutants, and water-related impacts (3). The experience of the National Health Service in England further shows that health systems can quantify their carbon footprint and use this information to guide decarbonization strategies across clinical services, procurement, travel, and infrastructure (4). These data are important for nursing because many opportunities for reducing waste and improving resource stewardship occur at the point of care, where nurses coordinate workflows, use consumables, educate patients, and maintain safety standards.

Gastrointestinal endoscopy is a particularly relevant setting for sustainable nursing practice. Endoscopy units are high-throughput environments characterized by frequent use of disposable accessories, sterile packaging, water-intensive reprocessing, chemical disinfectants, patient travel, and energy-consuming equipment. Recent literature reviews have emphasized that green endoscopy has developed from a general call for environmental awareness into an emerging field of evidence-based clinical practice (5, 6). Professional consensus has also advanced rapidly: the British Society of Gastroenterology, Joint Accreditation Group, and Centre for Sustainable Health have proposed practical measures across the endoscopy pathway, while the ESGE/ESGENA position statement has recommended embedding “reduce, reuse, and recycle” principles into endoscopy services and incorporating sustainability as a quality domain (7, 8).

International guidance has further reinforced the need for organizational change. The GI multisociety strategic plan from major US gastrointestinal societies called for coordinated action to reduce the environmental impact of digestive healthcare through sustainable clinical practice, education, research, and collaboration with industry (9). In the Asia-Pacific region, the APAGE position statements emphasized appropriate indications, careful planning of accessories, avoidance of unnecessary procedures, same-day combined procedures when appropriate, and the use of technology to improve efficiency (10). Together, these statements indicate that sustainable endoscopy is no longer simply an environmental aspiration; it is becoming an expected component of high-quality endoscopy service delivery. Empirical evidence has begun to define the scale of the problem and the potential for improvement. The Green Endoscopy Project Würzburg demonstrated that reducing unnecessary instruments, recycling packaging materials, and switching to alternative products can reduce Scope 3 carbon emissions without impairing endoscopic workflow (11). A prospective study of gastrointestinal procedures reported substantial solid waste, liquid waste, and energy consumption associated with routine endoscopy, while also identifying potentially recyclable materials that could be redirected through improved waste segregation (12). Similarly, a Korean audit quantified waste generation from gastrointestinal endoscopies and showed the need for local measurement before effective reduction strategies can be implemented (13). These studies suggest that sustainability interventions must be practical, measurable, and compatible with the pace and safety requirements of endoscopy units.

Despite these advances, a persistent implementation gap remains. Surveys of endoscopy healthcare professionals have shown that many staff recognize the importance of sustainable practice, but barriers such as limited training, lack of institutional support, uncertainty about waste streams, and absence of standardized metrics continue to hinder implementation (14). The recent E-SPARE position statement further highlighted the need for standardized reporting of environmental outcomes in endoscopy research, including material use, waste, energy, water, travel, and greenhouse gas emissions (15). For nursing, this means that sustainability cannot rely only on individual enthusiasm or informal “green champions”; it requires defined responsibilities, audit systems, education, and integration into service governance.

Within this implementation gap, the role of endoscopy nurses has been underdeveloped. Current discussions of green endoscopy often emphasize physician decision-making, such as indication control and avoidance of unnecessary procedures, or industry-led solutions, such as device redesign and alternative materials. These strategies are important, but they do not fully address the frontline processes through which waste and resource use are produced. Endoscopy nurses are directly involved in pre-procedure preparation, opening and managing consumables, assisting procedures, segregating waste, supporting bowel preparation, coordinating recovery, and participating in cleaning and reprocessing pathways. Repeated nursing micro-decisions therefore strongly influence whether sustainability guidance becomes routine practice or remains a policy-level statement.

Waste management is one of the clearest examples of nursing influence. The American Society for Gastrointestinal Endoscopy Task Force on Sustainable Endoscopy has emphasized appropriate management of endoscopic waste as a practical and scalable strategy for reducing the footprint of endoscopy units (16). However, correct waste segregation depends on the knowledge, confidence, and consistency of frontline staff, particularly nurses who handle packaging, procedure-related materials, and post-procedure disposal. When uncontaminated materials are incorrectly discarded as regulated medical waste, both environmental emissions and disposal costs increase unnecessarily. Conversely, accurate segregation and recycling can convert sustainability from an abstract institutional goal into a daily nursing quality-improvement activity. Key nursing-led and nursing-supported actions across the endoscopy pathway are summarized in Table 1.

The choice between single-use and reusable devices further illustrates the need for nursing participation in resource stewardship. Life-cycle and environmental impact studies have shown that single-use endoscopic equipment and endoscopes can generate substantial waste and carbon burdens, although infection-control considerations may justify their use in selected situations (17). More recent evidence comparing single-use and reusable gastroscopes similarly indicates that environmental effects vary according to context, reprocessing systems, and device-use assumptions (18). Nurses are not the sole decision-makers in device selection, but they are key contributors to risk assessment, pre-cleaning, leak testing, reprocessing compliance, documentation, and early identification of workflow problems that affect the safe and efficient use of reusable equipment. The selection of single-use versus reusable devices is nevertheless a clinical, infection-prevention, operational, and environmental decision that rests with the multidisciplinary team rather than with nursing staff alone.

Recent interventional evidence directly supports a nursing-centred approach. Menini et al. demonstrated that staff education, structured waste segregation, and the introduction of a Green Endoscopy nurse substantially reduced regulated medical waste, greenhouse gas emissions per procedure, and waste-management costs (19). A subsequent three-stage prospective study in Portugal similarly reported that awareness training focused on waste segregation and recycling significantly reduced the carbon footprint and waste-processing costs of endoscopic waste (20). This finding is particularly important for nursing because it shows that sustainability gains can be achieved through education, workflow redesign, role development, and audit, rather than through expensive technological change alone. It also reframes environmental responsibility as part of nursing quality and safety: appropriate waste disposal, resource stewardship, prevention of unnecessary repeat procedures, and support for efficient patient flow can protect both patients and the wider environment. However, the available evidence remains dispersed across studies of waste management, carbon accounting, life-cycle assessment, professional guidance, endoscope reprocessing, and service redesign. Few publications explicitly integrate these findings into a nursing practice framework that defines what endoscopy nurses should do, which competencies are required, how leadership should be organized, and which indicators can be used to evaluate improvement. This gap is important for contemporary nursing because the profession is increasingly expected to contribute not only to bedside care, but also to quality improvement, environmental responsibility, service redesign, and planetary health.

Therefore, this narrative review aims to construct a nursing-centred framework for sustainable endoscopy practice. Specifically, it examines how nurses can translate green endoscopy policy into clinical action across the endoscopy pathway, including waste segregation, reprocessing stewardship, consumable management, patient preparation, virtual follow-up, green procurement, sustainability education, and environmental audit. Because not all sustainability decisions in endoscopy are nursing-led, the framework explicitly distinguishes nursing-led actions from nursing-supported multidisciplinary decisions. By clarifying where nurses can lead and where nurses support multidisciplinary decision-making, this review seeks to clarify how nursing practice can reduce the environmental footprint of endoscopy while maintaining patient safety, procedural efficiency, and quality of care.

## Methods

This article was conducted as a structured narrative review and practice-oriented conceptual synthesis. The objective was not to generate pooled estimates of intervention effects, but to integrate current evidence, professional guidance, and implementation experience into a nursing-centred framework for sustainable endoscopy practice. The conduct and reporting of the review were informed by the Scale for the Assessment of Narrative Review Articles (SANRA) to improve clarity of the research question, search strategy, evidence selection, and synthesis process (21). A targeted literature search was conducted in PubMed/MEDLINE, Web of Science, Scopus, and Google Scholar from database inception through 31 August 2026. Search terms were developed around three core concepts: environmental sustainability, gastrointestinal endoscopy, and nursing practice. The main search terms included “green endoscopy”, “sustainable endoscopy”, “environmental sustainability”, “carbon footprint”, “medical waste”, “regulated medical waste”, “waste segregation”, “endoscope reprocessing”, “single-use endoscope”, “reusable endoscope”, “endoscopy nursing”, “nursing leadership”, “green nurse”, “healthcare sustainability”, and “planetary health”. Boolean combinations were adapted for each database. To ensure coverage of nursing-focused literature, a supplementary CINAHL search was conducted on 4 September 2026 using the same search concepts as the Scopus strategy, expressed in the CINAHL interface as: TX(“green endoscopy” OR “sustainable endoscopy” OR “green nurse” OR “green champion” OR “endoscopy nursing”) OR (TX(endoscop* OR colonoscop* OR gastroscop* OR duodenoscop*) AND TX(“environmental sustainability” OR “carbon footprint” OR “medical waste” OR “regulated medical waste” OR “waste segregation” OR “healthcare sustainability” OR “planetary health”)). Reference lists of key reviews, position statements, and consensus documents were also manually searched to identify additional relevant sources, particularly guidance from BSG/JAG/CSH, ESGE/ESGENA, US gastrointestinal societies, and APAGE (7–10).

Articles were considered eligible when they addressed environmental sustainability in gastrointestinal endoscopy or provided findings with clear implications for endoscopy nursing practice. Eligible sources included peer-reviewed original studies, prospective audits, life-cycle assessment studies, quality-improvement reports, implementation studies, narrative or practice-oriented reviews, professional position statements, and consensus documents. Particular attention was given to evidence related to waste segregation, resource use, endoscope cleaning and reprocessing, single-use versus reusable devices, patient preparation, service redesign, green procurement, sustainability education, and nursing leadership. Articles were excluded when they focused on environmental sustainability outside healthcare without transferable relevance to endoscopy practice, addressed endoscopy only from a purely technical or physician-centred perspective without implications for nursing workflow, or consisted solely of general commentary without practice-relevant content (5, 13, 22–26). The first and second authors screened the retrieved literature in two stages. First, titles and abstracts were reviewed for relevance to sustainable endoscopy and nursing practice. Second, potentially eligible full texts were assessed against the inclusion criteria. Disagreements regarding relevance or interpretation were resolved through discussion, and the corresponding author provided final adjudication and methodological oversight. Because this was a narrative review rather than a systematic review, formal meta-analysis and quantitative risk-of-bias scoring were not undertaken. However, priority was given to recent peer-reviewed studies, professional society statements, life-cycle analyses, prospective audits, and practice-based implementation reports.

The PubMed/MEDLINE, Web of Science, Scopus, and Google Scholar searches were updated and rerun through 31 August 2026 and identified 3,044 records. The supplementary CINAHL search conducted on 4 September 2026 identified 403 additional records before deduplication, bringing the total number of database records retrieved to 3,447. After cross-database deduplication, all 403 CINAHL records were found to duplicate records already present in the existing search set; therefore, the number of unique records remained 1,948. These 1,948 records were screened at title/abstract level, 86 reports underwent full-text assessment, 44 database-derived sources were retained, and six additional sources were identified through reference-list and professional-society searches, yielding 50 sources in the final narrative synthesis. No additional record from the supplementary CINAHL search proceeded to title/abstract screening, full-text assessment, or final inclusion. These counts are reported to improve transparency and do not imply that the review was conducted as a PRISMA-conformant systematic review. For each source, we distinguished (1) directly measured physical, resource, or activity outcomes (e.g., waste mass, water use, energy use, consumable counts, staff time, or costs), (2) calculated or modelled environmental estimates derived from measured activity data, emission factors, or life-cycle assessment, and (3) clinical or operational outcomes with plausible but unquantified environmental implications. Carbon-footprint values expressed as kg CO2e were treated as calculated or modelled outcomes unless a study directly measured greenhouse-gas flux; none of the included endoscopy studies did so. The third category was not interpreted as evidence of demonstrated environmental benefit.

Data were extracted and organized according to their relevance to nursing actions across the endoscopy pathway. The synthesis focused on six recurring domains: sustainability governance and nursing responsibilities, cleaning and reprocessing stewardship, waste segregation and consumable stewardship, full-pathway patient preparation and flow, multidisciplinary decision interfaces, and health-system context. To improve transparency, an evidence table was compiled summarizing the study design, setting, environmental endpoint, directness of environmental evidence, and degree of nursing involvement for the key included studies (Table 2). Evidence was then synthesized thematically to develop a nursing-centred framework that links specific nursing behaviours with environmental, economic, and operational outcomes. This approach allowed the review to move beyond a general description of green endoscopy and to clarify how nurses can translate sustainability policy into daily clinical practice.

**Table 2:** Key evidence informing this review: study design, environmental endpoint, evidence directness, and nursing involvement.

Author, year, country	Study design	Unit/procedure scope	Environmental endpoint	Direct/Modelled/Inferred	Nursing involvement	Key finding
Namburar et al., 2022, USA (17)	Five-day cross-sectional waste audit with estimation	Two academic endoscopy centers	Waste mass; modelled waste scenarios	Direct (waste) + modelled	Not nursing-specific	Quantified disposable waste per procedure and estimated waste implications of transitioning to single-use endoscopes; not a full LCA.
Desai et al., 2024, USA (12)	Prospective audit	Endoscopy unit procedures	Solid/liquid waste, energy	Direct	Not nursing-specific	Substantial solid waste, liquid waste, and energy consumption per procedure; identified recyclable materials.
Jung et al., 2025, South Korea (13)	Waste audit	GI endoscopies	Waste mass; estimated carbon footprint	Direct + modelled	Not nursing-specific	Quantified waste generation; local measurement needed before reduction strategies.
Henniger et al., 2024, Germany (11)	Prospective intervention (Green Endoscopy Project Würzburg)	Endoscopy unit	Scope 3 carbon emissions	Direct activity/product-use data + calculated/modelled carbon footprint	Multidisciplinary	Reduced Scope 3 emissions without impairing endoscopic workflow.
Cunha Neves et al., 2023, Portugal (22)	Targeted quality-improvement intervention	GI endoscopy unit	Waste mass	Direct	Multidisciplinary	Targeted intervention achieved measurable waste reduction.
Menini et al., 2026, Italy (19)	Prospective intervention	Endoscopy unit (Humanitas Research Hospital)	RMW mass, kgCO2e per procedure, cost	Direct (RMW mass, cost) + calculated/modelled (kg CO2e)	Nursing-sensitive; Green Endoscopy nurse monitored compliance	RMW −24.1%; calculated GHG per procedure −33.3% (−1.23 kg CO2e); ∼€3,542 saved in 3 months.
Dias Blanco et al., 2026, Portugal (20)	Three-stage prospective before–after study	Endoscopy unit (Santa Maria Hospital)	Carbon footprint, waste-processing costs, waste mass	Direct (waste mass, cost) + calculated/modelled (kg CO2e)	Nurse-relevant (education and segregation)	Carbon footprint calculated from waste mass using predefined emission factors; 1859.5→1334.8 kg CO2e and costs USD 302.1→208.1 after intervention.
Hicks & Mutowo, 2025, UK (35)	Observational time-and-motion study with validated SOP redesign	Endoscope decontamination unit	Water use, cleaning time	Direct (per endoscope) + modelled (annual)	Nurse-led	−25 L water per manual cleaning; ∼2 min saved per endoscope; estimated 725,000 L water saved annually.
Pioche et al., 2024, France (18)	ISO 14040 life-cycle assessment	Gastroscope (device level)	kgCO2e per examination	Modelled (LCA)	Not nursing-specific	Single-use gastroscope 10.9 vs. reusable 4.7 kg CO2e per examination.
Welsch & Eickhoff, 2026, Germany (29)	ISO-compliant cradle-to-grave LCA	Duodenoscope per ERCP (device level)	kgCO2e per ERCP	Modelled (LCA)	Not nursing-specific	Single-use 6.61 vs. reusable 3.98 kg CO2e per ERCP under German reprocessing conditions.
Gayam et al., 2026, USA (45)	Prospective one-week audit with LCA, three centers	Whole routine endoscopic procedure	kgCO2e per procedure	Direct activity data + calculated/modelled (LCA)	Multidisciplinary	Mean 53.2 kgCO2e per procedure; HVAC/electricity 42%, patient travel 29%, supplies 22%, reprocessing 4%; same-day EGD + colonoscopy −42%.
Lämmer et al., 2025, Netherlands (44)	Single-center LCA	Colonoscopy	kgCO2e per colonoscopy	Modelled (LCA)	Not nursing-specific	56.4 kgCO2e per colonoscopy; transport of patients and staff dominant (76.5%).
García-Castellanos et al., 2026, Spain (47)	Prospective multifactorial footprint assessment with sensitivity analysis	Diagnostic colonoscopy	kgCO2e per colonoscopy	Modelled (cradle-to-grave LCA)	Not nursing-specific	18.09 kgCO2e in base scenario; patient and staff transport the main contributors.
Vegting et al., 2026, Netherlands (46)	Systematic review (28 studies)	GI endoscopy services	kgCO2e and other impact categories	Mixed	Not nursing-specific	Per-procedure emissions 5.4–73.2 kgCO2e; travel, energy, and products dominate; substantial methodological heterogeneity.
Leddin et al., 2026, Canada/USA (32)	Society framework (CAG/ASGE)	Endoscopy services	Sustainability indicators	Not applicable	Nursing-relevant	Seven indicator categories: administration, education, appropriateness, consumables, waste, travel, and energy.
Rughwani et al., 2025, India (48)	Prospective multi-dimensional assessment	3,873 GI endoscopy procedures at a tertiary center	Waste, travel, electricity, water, gases; kg CO2e	Direct activity/waste data + calculated emissions	Not nursing-specific	38.45 kg CO2e/procedure including travel; travel 83.09%, electricity 10.42%; illustrates context outside predominantly high-income evidence.
Izci et al., 2026, Netherlands (41)	Single-center material-flow analysis	15 diagnostic colonoscopies	Waste mass/material composition; estimated waste carbon footprint	Direct (material/waste) + calculated/modelled (kg CO2e)	Nursing-relevant at preparation/consumable stage	Mean waste 0.58 kg/procedure; many products used/discarded in preparation stage; waste carbon footprint estimated at 1.77 kg CO2e.
Olabintan et al., 2026, multinational cohort (50)	Carbon-footprint assessment using prospective colonoscopy cohort outcomes	1,134 patients; CADx versus no CADx under leave-in-situ strategy	kg CO2e from devices, consumables, and histopathology	Prospective clinical activity data + calculated/modelled LCA	Not nursing-specific	Despite fewer snares/polyp traps and histopathology submissions, CADx increased the modelled footprint by 0.10 kg CO2e per colonoscopy.

In this table, kg CO2e values are treated as calculated/modelled environmental outcomes unless greenhouse-gas flux was directly measured; none of the listed endoscopy studies directly measured greenhouse-gas flux.

## Defining and institutionalizing sustainability responsibilities: nursing governance and resource stewardship

With the market promotion of single-use endoscopes, endoscopy nursing faces a continuing challenge in balancing infection control and environmental sustainability (27, 28). Single-use endoscopes avoid reprocessing-related cross-contamination attributable to reuse, but their environmental burden should be quantified rather than assumed (28). Namburar et al. conducted a five-day cross-sectional waste audit at two US academic centers and estimated the waste implications of transitioning from reusable to single-use endoscopes; this was not a full life-cycle assessment (17). In contrast, Pioche et al. performed an ISO 14040 life-cycle assessment and reported a higher per-examination carbon footprint for the evaluated single-use gastroscope than for the reusable gastroscope (10.9 vs. 4.7 kg CO2e) (18). More recently, an ISO-compliant cradle-to-grave life-cycle assessment conducted under German reprocessing conditions reported higher per-procedure emissions for a single-use duodenoscope than for a reusable duodenoscope (6.61 vs. 3.98 kg CO2e per ERCP) (29). These device-level results are modelled estimates that depend on device type, lifetime assumptions, local energy mix, and reprocessing infrastructure, and should not be generalized uncritically.

Nursing staff are directly involved in maintaining the integrity and safe reuse of reusable endoscopes. Standardized bedside pre-cleaning and leak testing are important components of safe reprocessing and device care (23, 30). Where safe device longevity is extended, replacement-related material demand may be reduced, but the environmental magnitude of that downstream effect has not been directly quantified in the cited studies.

The selection of single-use versus reusable endoscopes is a clinical, infection-prevention, operational, and environmental decision that should be governed by multidisciplinary policy rather than by nursing staff alone. Nurses contribute by identifying infection-control concerns during pre-procedure assessment, ensuring correct pre-cleaning and reprocessing of reusable devices, documenting failures or barriers in the reprocessing pathway, and providing operational information to endoscopists, infection-prevention teams, and procurement leads. An international Delphi consensus has framed single-use endoscopy adoption as a staged, indication-driven process rather than routine use (31), and the ESGE/ESGENA position statement recommends embedding reduce–reuse–recycle principles at the service level (8). Within such governance, standardized waste measurement systems, such as the audit framework used by Jung et al. in Korea, can inform unit-level policy (13).

To make these responsibilities sustainable, it has been proposed that units move beyond a “volunteer model” reliant on individual enthusiasm. Donnelly has called for institutionalized “Green Nurse” or “Green Champion” functions and their integration into accreditation frameworks such as JAG (23). In this review, institutionalization refers to making sustainability responsibilities explicit within nursing governance—defined responsibilities, education, audit, and feedback—rather than asserting that every unit must create a new mandatory position. A designated Green Endoscopy Nurse or sustainability-lead function may be considered as an implementation strategy in larger endoscopy units, particularly to coordinate staff education, waste auditing, feedback, and sustainability metrics (19, 20). Current evidence is promising but limited, and does not yet establish this role as a universal staffing standard. Where indicators are adopted, recently proposed society-level frameworks—such as the CAG/ASGE sustainability indicators spanning administration, education, appropriateness, consumables, waste, travel, and energy (32)—and the E-SPARE reporting recommendations (15) provide more standardized options than unit-invented metrics. Nursing staff are therefore not only maintainers of daily operations in the endoscopy unit but also important operational contributors to the transition from a “disposable culture” toward a more circular use of resources.

## Cleaning, reprocessing, and resource stewardship within validated safety protocols

Beyond solid waste management, the endoscope reprocessing process is another hotspot for resource consumption, particularly regarding water and chemical reagent usage; reprocessing has been identified as a relevant source of resource consumption and emissions in gastrointestinal endoscopy (33). Traditional endoscope cleaning workflows are not only water-intensive but also occupy a significant amount of nursing time (33). In this domain, nurses are not just operators but contributors to process optimization (34). Any process simplification must remain subordinate to validated reprocessing protocols, infection-prevention requirements, and local regulatory compliance.

An empirical study by Hicks and Mutowo (2025) at Blackpool Teaching Hospitals in the UK illustrates process optimization within a validated reprocessing pathway (35). The investigators conducted a single-center direct observational time-system analysis before and after replacement of the standard detergent with EndoPreZyme™, with the final manual rinse omitted in accordance with the detergent instructions for use when immediate processing in a validated endoscope washer-disinfector followed. Water consumption was reduced by 25 liters per endoscope; based on the projected annual workload, the authors estimated a potential saving of 725,000 liters of water per year (35).

The same study reported operational co-benefits: manual cleaning time per endoscope fell by an average of about 2 min, which the authors estimated could release approximately 962.9 h of nursing or technician time annually in a high-volume center, freeing staff capacity for patient care or other value-added activities (35). These findings are consistent with Donnelly's observation that careful management of cleaning, disinfectant use, and equipment maintenance by nursing staff can extend equipment lifespan and reduce chemical waste (23, 36). Together, these studies suggest that environmental and efficiency goals can be aligned in reprocessing practice, providing practical arguments for nursing managers seeking institutional support for sustainability projects.

Additionally, high-level disinfection of endoscopes relies on potent chemicals such as glutaraldehyde and peracetic acid, which can pose occupational and environmental hazards (37). Within validated protocols, careful monitoring of disinfectant concentration and replacement intervals may help avoid premature disposal of usable solution (36, 38). Nursing managers can also contribute to multidisciplinary procurement discussions by requesting evidence on safety, resource use, occupational exposure, and life-cycle impact when alternative disinfection technologies are considered. For example, UV-C has been evaluated for bacterial-reduction efficacy in a randomized controlled trial (39), but that study did not assess environmental outcomes; environmental superiority should therefore not be inferred from disinfection efficacy alone.

## Nursing-led waste segregation, education, and consumable stewardship

Among the implementation pathways of green endoscopy, waste segregation and consumable stewardship are among the most directly studied areas in which nursing teams can lead. Medical waste should be separated into general waste and regulated medical waste (RMW) according to local infection-control and waste-management requirements. In high-pressure clinical environments, convenience bias or defensive over-caution regarding infection can lead staff to discard substantial amounts of uncontaminated packaging and paper into higher-treatment waste streams (40). Such over-segregation increases disposal burden and, where regulated waste is treated through more carbon-intensive pathways, can increase calculated emissions and processing costs (22, 23).

Addressing this problem, Menini et al. (2026) conducted a prospective intervention study at Humanitas Research Hospital in Italy (19). The intervention included staff education, dedicated paper and plastic recycling bins, and a Green Endoscopy nurse who monitored compliance. RMW generation decreased by 24.1%, and the calculated GHG emissions associated with RMW decreased by 33.3% (net difference 1.23 kg CO2e per procedure); waste-management costs were reduced by approximately €3,542 over the three-month study period (19). A subsequent three-stage prospective study at Santa Maria Hospital in Portugal reported convergent findings after awareness training focused on waste segregation and recycling: waste-processing costs fell from USD 302.1 to USD 208.1, while the reported carbon footprint of endoscopic waste fell from 1859.5 to 1334.8 kg CO2e (20). In that study, carbon footprint was calculated from waste mass using predefined emission factors rather than directly measured as greenhouse-gas flux.

These prospective studies suggest that re-engineering nursing workflows and standardizing staff behavior—such as separating and recycling outer packaging during patient set-up (12)—can align environmental and economic goals within the study settings. A 2026 material-flow analysis of diagnostic colonoscopy also found that many products were used and discarded during the preparation stage, supporting targeted review of consumable selection and opening practices (41). Donnelly (2022), in his discussion on green endoscopy implementation, similarly emphasizes recycling processes and accurate waste-stream identification as operational priorities (23, 24). Consumable stewardship is therefore a related nursing-led domain: avoiding premature opening of accessories, confirming procedure type and anticipated device needs before sterile packs are opened, and recording unused opened items as part of quality improvement can reduce avoidable single-use consumption (12, 16, 41). Taken together, the evidence supports a direct nursing role in waste segregation and consumable management while avoiding attribution of the entire procedural carbon footprint to nursing behavior alone.

## Full-pathway nursing contributions and multidisciplinary interfaces

Preventing avoidable repeat examinations is a plausible pathway to reducing resource use. When colonoscopy must be repeated because of inadequate bowel preparation, an additional episode of consumables, reprocessing, staff time, and patient travel is required. Nurse-led telephone follow-ups, SMS reminders, and digital education tools have been shown to improve bowel-preparation adequacy (42). However, the cited studies measured preparation quality and repeat rates rather than environmental outcomes; the environmental benefit of preparation support is therefore plausible but was not directly quantified. Protocol optimization may further improve adherence and reduce repeat procedures, but direct carbon savings require procedure-level environmental evaluation.

Reducing avoidable patient travel—a relevant component of Scope 3 emissions—is another plausible nursing-supported pathway. Nurses can support virtual pre-procedure assessment or post-procedure follow-up when clinically appropriate, including preparation guidance and communication of results (43). The virtual-care literature cited here did not directly quantify endoscopy-related carbon savings, so this pathway is treated as indirect. Its potential relevance is nevertheless context dependent: in a Dutch colonoscopy life-cycle assessment, transport of patients and staff was the dominant contributor (44), and in a three-center US assessment patient travel accounted for 29% of the calculated procedure footprint (45).

Coordinating esophagogastroduodenoscopy (EGD) and colonoscopy on the same day, when clinically appropriate, can reduce duplicated resource use. In a three-center US assessment using prospective activity data and life-cycle modelling, same-day EGD and colonoscopy for the same patient was associated with a 42% lower combined calculated carbon footprint than performing the procedures separately (45). This is a modelled whole-procedure environmental estimate rather than direct greenhouse-gas measurement. Scheduling decisions of this kind remain multidisciplinary, balancing clinical indication, sedation considerations, patient preference, and unit capacity; nurses contribute through coordination, preparation, and patient education rather than independent decision-making (7, 10).

## Context-specific implementation: health-system resources, energy mix, and the distribution of endoscopy-related emissions

The environmental priorities of an endoscopy unit are context dependent. Cross-country comparisons are complicated by differences in system boundaries, national electricity generation, patient and staff travel, waste-treatment pathways, reprocessing technology, and the intensity of single-use consumables. A 2026 systematic review found substantial methodological heterogeneity, including inconsistent Scope 3 accounting and country-specific electricity emission factors, with calculated per-procedure estimates ranging from 5.4 to 73.2 kg CO2e (46). Recent procedure-level studies illustrate this variability: transport of patients and staff dominated a Dutch colonoscopy LCA (76.5%) (44), and a Spanish multifactorial assessment likewise identified travel as the main contributor (47), whereas a three-center US study found heating, ventilation, air conditioning, and electricity to be the largest source (42%), followed by patient travel (29%), supplies (22%), and reprocessing (4%) (45). Evidence from outside the high-income settings that dominate the literature also illustrates contextual variation: a large prospective study from an Indian tertiary center reported a calculated footprint of 38.45 kg CO2e per procedure including patient travel, with patient travel contributing 83.09%, electricity 10.42%, medical gases 3.63%, and water 1.86% (48). These findings support local measurement rather than uncritical transfer of a single intervention hierarchy across settings. Direct comparative evidence by national income group remains limited, so these differences should not be interpreted as an established income-gradient effect.

It is also useful to distinguish the whole-procedure footprint from the domains that nursing practice can influence. The whole-procedure footprint includes facility energy and HVAC, patient and staff travel, supplies and instruments, reprocessing, waste treatment, and sedation or anesthesia (45, 46, 48). Nursing-sensitive domains include waste segregation, consumable opening and stocking, reprocessing compliance, patient education, audit and feedback, and aspects of travel-reduction coordination. No included study isolated a nursing-attributable percentage of whole-procedure kg CO2e; Figure 1 therefore maps domains of direct, shared, and limited nursing influence rather than assigning a proportion of total greenhouse-gas emissions to nursing practice.

**Figure 1:**
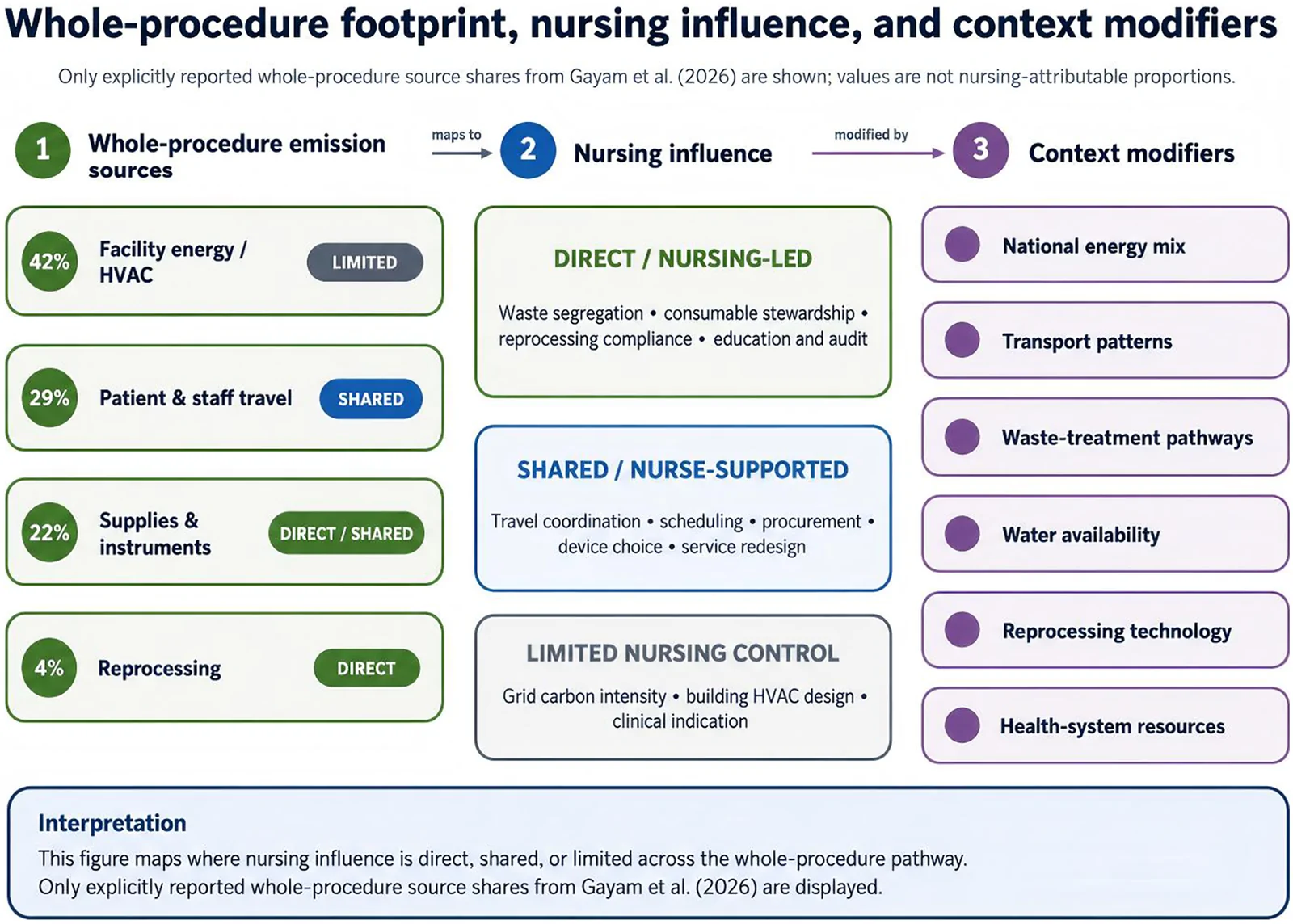
Whole-procedure environmental footprint, nursing-sensitive domains, and context modifiers in gastrointestinal endoscopy. The relative contribution of emission sources varies by local energy mix, travel patterns, waste treatment, reprocessing systems, and study boundaries. The numerical proportions shown are from Gayam et al. (2026) and represent an illustrative three-center US example rather than a universal distribution. These proportions are whole-procedure source shares and should not be interpreted as the percentage of emissions attributable to nursing practice.

## Implications for nursing practice

The findings of this review have several implications for endoscopy nursing practice. First, environmental sustainability should be incorporated into routine nursing quality and safety work rather than being treated as an optional or peripheral activity. Endoscopy nurses are positioned at the point where clinical decisions become operational actions, including patient preparation, consumable use, waste segregation, recovery coordination, and reprocessing support. Therefore, planetary health literacy should be regarded as part of contemporary nursing competence, alongside infection prevention, patient safety, and quality improvement (1, 2). The nursing-centred framework proposed in this review is summarized in Figure 2.

**Figure 2:**
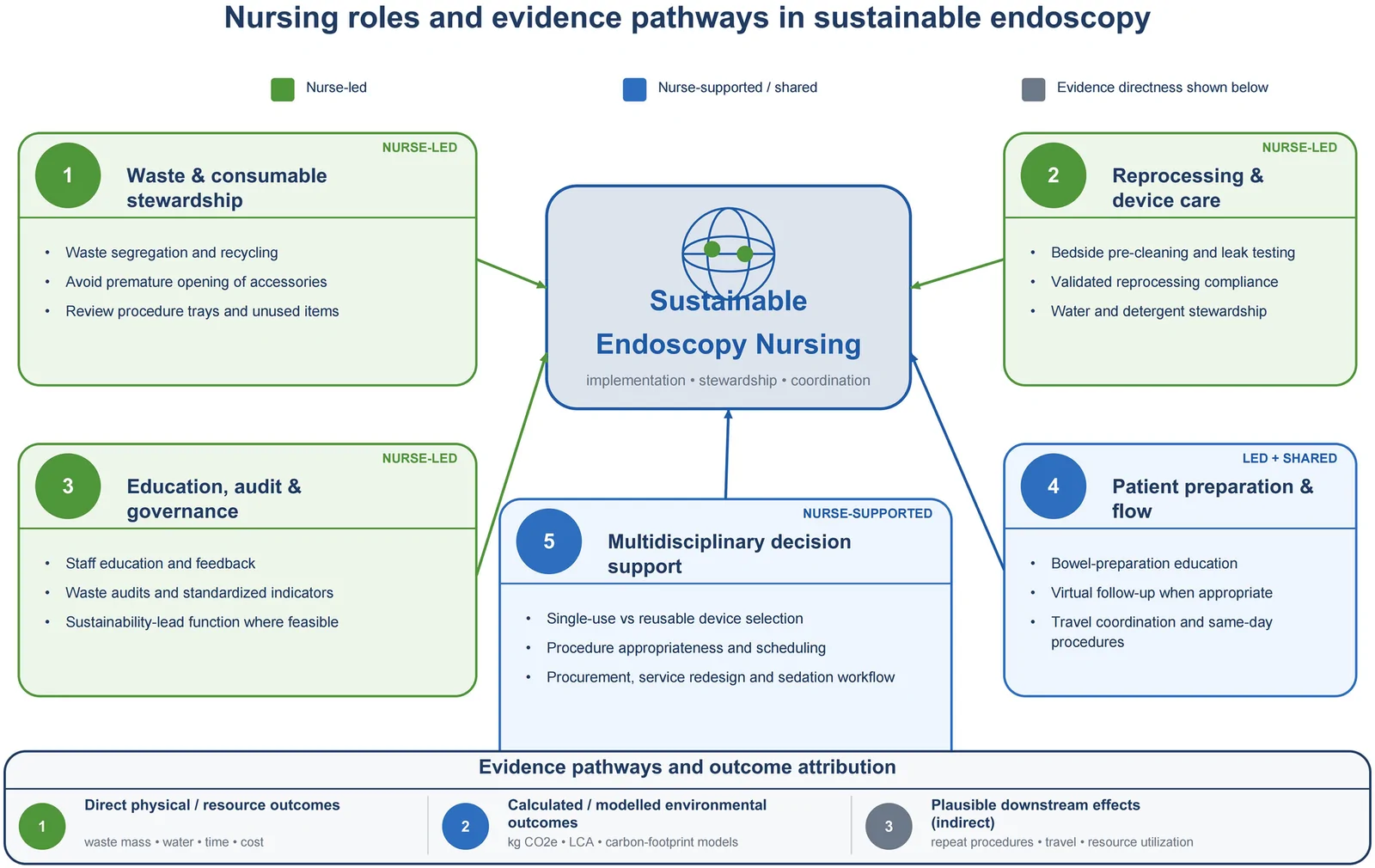
Nursing roles and evidence pathways in sustainable endoscopy. The framework distinguishes nursing-led actions from nursing-supported multidisciplinary decisions and separates directly measured physical/resource outcomes from calculated/modelled environmental outcomes and plausible but unquantified downstream effects. It is an attribution map rather than a causal model; downstream outcomes should not be interpreted as directly caused by nursing practice unless supported by the corresponding evidence category.

Second, endoscopy units should translate broad green endoscopy recommendations into practical nursing protocols. At the pre-procedure stage, nurses can reduce avoidable resource use by confirming procedure type, anticipated accessories, patient readiness, and bowel preparation status before sterile items are opened. Nursing-led education through written instructions, telephone reminders, digital tools, or pre-assessment calls can improve preparation adequacy; the associated environmental benefit is plausible but has not been directly quantified in the cited studies. These actions are consistent with current regional guidance, which emphasizes appropriate procedures, efficient scheduling, and avoidance of unnecessary endoscopic interventions as key components of sustainable gastrointestinal practice (10).

Third, waste segregation should become a core nursing workflow rather than an informal environmental behaviour. Clear separation of regulated medical waste, general waste, and recyclable clean packaging should be embedded into local standard operating procedures, supported by visible bin labelling, staff education, and periodic waste audits. This is a practical area in which nurses can generate measurable environmental and economic benefits without compromising infection control. Recent guidance on endoscopic waste management supports such unit-level changes, and prospective evidence has shown that staff education and structured waste segregation can reduce regulated medical waste, associated greenhouse gas emissions, and waste-processing costs in high-volume endoscopy practice (16, 19, 20).

Fourth, nursing practice should contribute more actively to resource stewardship during cleaning and reprocessing. Although reprocessing protocols must always prioritize patient safety and regulatory compliance, nurses can help reduce unnecessary resource use by ensuring timely bedside pre-cleaning, careful handling of reusable scopes, accurate leak testing, correct documentation, and early reporting of equipment damage or process inefficiencies. Reprocessing is a resource-intensive component of endoscopy care, involving water, detergents, disinfectants, energy, and staff time; therefore, nursing attention to process reliability can support both sustainability and equipment longevity (33, 36).

Fifth, a designated Green Endoscopy Nurse or sustainability-lead function may be considered in larger endoscopy units. This function may include staff education, waste-stream auditing, feedback to the clinical team, review of high-use consumables, participation in procurement discussions, and coordination of sustainability indicators. Candidate indicators are increasingly standardized—for example, the CAG/ASGE framework spanning administration, education, appropriateness, consumables, waste, travel, and energy (32)—and the E-SPARE position statement provides a basis for standardized environmental reporting (15). Current evidence supports the Green Endoscopy Nurse as a promising implementation model rather than an established staffing standard (19). Nursing input is also valuable in green procurement and service redesign, but these remain multidisciplinary responsibilities (36, 49). Sustainability education may be incorporated into endoscopy nursing orientation, competency assessment, and continuing professional development, consistent with broader nursing planetary-health literature and emerging endoscopy indicator frameworks (1, 2, 15, 32). Training can focus on waste segregation, preventing unnecessary opening of devices, preparation support, audit documentation, and escalation of system barriers. These recommendations represent a practice framework rather than a universally validated competency standard.

## Evidence gaps and future research

The evidence base for sustainable endoscopy nursing remains limited in several ways. First, few intervention studies are nursing-specific: most environmental studies measure departmental or procedural footprints without isolating nursing contributions (46). Second, methodological heterogeneity—including inconsistent system boundaries and Scope 3 accounting—limits cross-study and cross-country comparability, and the evidence remains concentrated in a relatively small number of settings (45, 46, 48). Third, standardized nursing-sensitive sustainability indicators are only beginning to emerge (15, 32). Recent direct environmental evidence also cautions against assuming that digital innovation is inherently ‘green': a 2026 carbon-footprint assessment of computer-aided diagnosis in colonoscopy found that CADx reduced some consumable and histopathology use but increased the total modelled footprint by 0.10 kg CO2e per colonoscopy (50). Future work should prospectively evaluate nursing-sensitive sustainability indicators, report directly measured resource outcomes alongside transparent carbon-footprint methods, and examine how local energy mix, travel patterns, waste infrastructure, and reprocessing systems modify the impact of sustainability interventions.

## Conclusion

This narrative review suggests that the most directly actionable nursing contributions to sustainable endoscopy lie in waste segregation, consumable stewardship, reliable reprocessing practice, staff education, audit, and implementation of unit-level sustainability policies. Prospective studies show that structured education and waste segregation can reduce regulated medical waste and processing costs, with associated reductions in calculated or modelled carbon footprints (19, 20), while recent life-cycle assessments highlight context-dependent environmental trade-offs between reusable and single-use devices (18, 29). For decisions involving procedure indication, sedation, procedure timing, combined procedures, and device selection, nursing input should be framed as supportive within multidisciplinary governance rather than as independent clinical decision-making.

The evidence base remains heterogeneous and is concentrated in high-resource settings, with relatively few nursing-specific intervention studies and limited standardization of environmental outcomes (32, 46). A designated Green Endoscopy Nurse or sustainability-lead function is therefore best viewed as a promising implementation model rather than an established standard. Future work should prospectively evaluate nursing-sensitive sustainability indicators, report direct environmental outcomes using standardized frameworks, and examine how local energy mix, travel patterns, waste infrastructure, and reprocessing systems modify the impact of endoscopy sustainability interventions.
